# Effects of 75 min of weekly exercise snacks on body composition, cardiometabolic health, cardiorespiratory fitness, and lower-body explosive power in overweight and obese female university students

**DOI:** 10.3389/fpubh.2026.1846870

**Published:** 2026-07-15

**Authors:** Naijing Jin, Xin Zheng, Ziren Zhao, Fangyuan Zhao, Zhangyuting Shang, Kaixiang Zhou

**Affiliations:** 1 Journal Editorial Department, China Institute of Sport Science, Beijing, China; 2 College of Physical Education and Health Science, Chongqing Normal University, Chongqing, China; 3 Sports Coaching College, Beijing Sport University, Beijing, China

**Keywords:** body composition, exercise snacks, female, high-intensity interval training, overweight and obese

## Abstract

**Background:**

Overweight and obesity have become increasingly prevalent among female university students, whereas conventional exercise interventions are often limited by poor adherence and time constraints. Exercise snacks (ES) may offer a flexible and time-efficient alternative; however, their effectiveness in this population remains unclear.

**Objective:**

We compared the effects of 8 weeks of ES (75 min/week) and HIIT on body composition, cardiometabolic health, cardiorespiratory fitness, and lower-body explosive power in overweight and obese females in a real-world setting.

**Methods:**

Thirty overweight and obese female university students were randomized to either the ES group or the high-intensity interval training (HIIT) group in an 8-week randomized controlled trial. Both groups completed 75 min of bodyweight exercise per week. Pre- and post-intervention assessments included body composition, cardiometabolic health, cardiorespiratory fitness, and lower-body explosive power. A two-way repeated measures ANOVA was used to analyze training effects, with Bonferroni *post hoc* tests. Effect sizes were reported as partial eta squared ( $\eta_{p}^{2}$ ) or Cohen’s d, with significance set at *p* < 0.05.

**Results:**

Compared with the HIIT group, the ES group showed significantly lower RPE throughout the intervention period (*p* < 0.05). Significant time effects were observed for body weight (*p* < 0.001, $\eta_{p}^{2}$ = 0.59), BMI (*p* < 0.001, $\eta_{p}^{2}$ 0.60), body fat percentage (*p* < 0.001, $\eta_{p}^{2}$ = 0.46), standing long jump performance (*p* < 0.001, $\eta_{p}^{2}$ = 0.43), V̇O_2max_ (*p* < 0.001, $\eta_{p}^{2}$ = 0.47), SBP (*p* < 0.001, $\eta_{p}^{2}$ = 0.54), TC (*p* = 0.004, $\eta_{p}^{2}$ = 0.26), and LDL-C (*p* = 0.002, $\eta_{p}^{2}$ = 0.30). A significant time × group interaction was observed for DBP (*p* = 0.02, ηp^2^ = 0.19), with a significant reduction in the HIIT group (*p* = 0.002, d = 0.86) but not in the ES group (*p* = 0.86, d = −0.04). No significant time effects were observed for TG (*p* = 0.96) or HDL-C (*p* = 0.10). Weekly mean RPE was significantly lower in the ES group than in the HIIT group throughout the intervention period.

**Conclusion:**

In conclusion, in a real-world remote setting, 75 min/week of bodyweight-based ES may be a promising strategy that warrants confirmation in adequately powered randomized controlled trials.

## 1 Introduction

Obesity has become a global health problem, contributing to complications such as hypertension, diabetes, coronary heart disease, and atherosclerosis. A recent analysis of 3,663 studies showed that the global prevalence of obesity among adult females rose from 8.8% in 1990 to 18.5% in 2022 (1). University students represent a particularly high-risk population for overweight and obesity, with evidence suggesting that approximately 22% of university students worldwide are classified as overweight or obese (2). Insufficient physical activity is a major contributor to the obesity epidemic among university students (3). The World Health Organization (WHO) guidelines recommend that adults engage in at least 150 min of moderate-intensity or 75 min of vigorous-intensity exercise each week; however, many individuals fail to meet these recommendations (4). A major reason university students struggle to meet physical activity guidelines is time constraints. University students often struggle to develop exercise habits due to academic pressure, lack of motivation, and insufficient social support (5, 6). This poses a certain challenge to the implementation of regular exercise (e.g., 30 min or more, 2–3 times per week), particularly for physically inactive and sedentary students. For example, high-intensity interval training (HIIT) has consistently had low participation rates due to high time requirements and intense exertion (7). Therefore, developing a safe, flexible, and time-efficient exercise model is of considerable importance for promoting physical activity and improving the health of overweight and obese university students.

Exercise snacks as a novel, lifestyle-integrated exercise (e.g., stair climbing) may offer a promising solution to the limitations mentioned above, due to its time efficiency, flexibility in scheduling, operational safety, enjoyment of the exercise, and increased motivation for participation (8, 9). Exercise snacks involve breaking down a single continuous exercise duration (e.g., 75 min) into multiple short bursts of activity (1–10 min) spread out throughout the day, to promote health (10). Zhou et al. (11) showed that implementing ES can effectively reduce visceral abdominal fat and epicardial adipose tissue in obese adults. Rafiei et al.’s (12) studies have demonstrated significant improvements in postprandial insulin and non-esterified fatty acid levels among obese adults. Additionally, previous studies have shown that ES improves aerobic capacity (13, 14), muscular strength (15), and metabolic health (12), while also demonstrating high acceptability and adherence (16), making it suitable for individuals with limited time or low motivation for exercise. Nevertheless, some studies have been conducted in laboratory or tightly controlled settings (13, 17), with few studies investigating the feasibility and efficacy of bodyweight-style exercise snacks in the real world (18), especially for overweight and obese females.

Additionally, the dosage of ES is unclear. At present, the weekly exercise volume for ES does not meet WHO recommendations (e.g., at least 75 min of vigorous-intensity physical activity per week) (4, 13, 14, 19–21). For example, Wong et al. (22) found that a six-week sprint cycling program, involving a daily 30-s effort for five days a week (150 s total), did not improve cardiorespiratory fitness or strength in active young adults. Fyfe et al. (16) also reported that a four-week home-based resistance program was safe and feasible for older adults, but it did not significantly enhance performance on the 5-times sit-to-stand or 30-s sit-to-stand tests compared to a control group. The underlying reason for these unfavorable outcomes is insufficient total weekly exercise.

This study employed smart remote exercise devices in a real-world setting to evaluate the effects of an 8-week ES intervention, totaling 75 min per week, compared with HIIT, on body composition, cardiometabolic health, cardiorespiratory fitness (CRF), and lower-body explosive power in overweight and obese females. We hypothesized that, compared with the HIIT group, the ES intervention would confer comparable benefits on body composition, cardiometabolic health, cardiorespiratory fitness, and lower-body explosive power, while eliciting lower ratings of perceived exertion (RPE).

## 2 Materials and methods

### 2.1 Participants

The sample size was informed by previous exercise-snacking (*n* = 13) (21, 23). In addition, a power analysis was conducted using G*Power version 3.1.9.7, with parameters based on commonly used standards in related studies, including a medium effect size (*f* = 0.25), *α* = 0.05, statistical power (1-*β*) = 0.80, and a correlation among repeated measures of r = 0.50 (24). The analysis indicated that 34 participants were required to detect a group-by-time interaction. Due to the exploratory nature of this study and the challenges of implementing a real-world intervention, 30 participants were ultimately recruited. Because the final sample size was below the *a priori* estimate, the present findings should be interpreted as preliminary and exploratory.

A total of 30 overweight and obese female college students, with a BMI greater than 25 kg/m^2^, participated in this study (25, 26). Participants were free of cardiovascular, pulmonary, neurological, metabolic, or orthopedic disorders. To minimize the impact of menstrual irregularities, participants with irregular cycles were excluded. The study was approved by the Ethics Committee of China Institute of Sport Science (No. 20250408), and all procedures were conducted in accordance with the Declaration of Helsinki. Before the experiment, participants were informed of the benefits and potential risks related to the study, and all signed the informed consent form.

### 2.2 Study design

**Figure 1:**
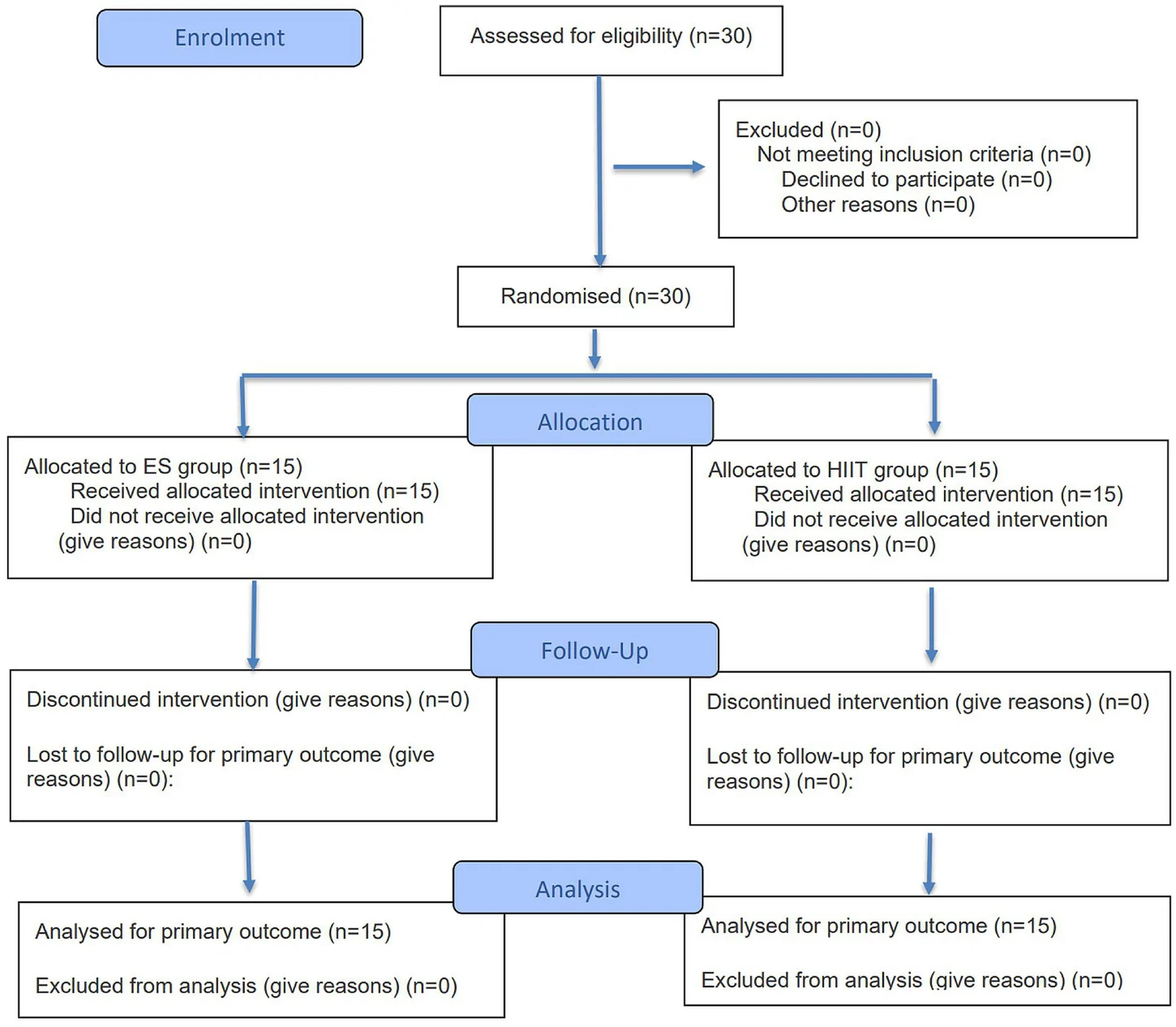
CONSORT 2025 flow diagram.

This study used an eight-week randomized controlled trial (Figure 1). Participants were randomly assigned to either the ES (*n* = 15) or the HIIT (*n* = 15) group using a computer-generated block randomization sequence (block size = 4). The allocation sequence was generated by an investigator not involved in participant recruitment or assessment, and the assignments were concealed until interventions were implemented. Due to the nature of the exercise interventions, participants and intervention supervisors could not be blinded to group allocation. The outcome assessor was blinded to group assignments and was not involved in recruiting, randomizing, or supervising training. The assessor had no access to training records or group information, and participants were instructed not to reveal their group assignment during assessments. Participants were instructed to maintain their usual lifestyle and dietary habits throughout the intervention period and to refrain from any additional structured exercise training outside the prescribed protocol to minimize potential confounding effects. All participants completed outcome measurements before (baseline) and after the intervention. Participants were familiarized with the testing procedures and training program one week before baseline testing. Demographic information and anthropometric data (weight and height) were recorded. To ensure measurement consistency and minimize potential bias, all evaluations were conducted by trained outcome assessors who were blinded to group allocation and followed standardized testing procedures. All evaluation sessions were conducted at the same time of day for each participant to prevent potential confounding effects related to circadian rhythms. Temperature (21.2 ± 0.3 °C) and humidity (29.0 ± 0.4%) were maintained consistently. Participants were instructed to avoid vigorous exercise for 48 h before each evaluation day and refrain from consuming alcohol and caffeine for 24 h before the sessions.

### 2.3 Intervention protocol

To accommodate students’ course schedules, the intervention was delivered remotely in a real-world setting using a smart exercise app, with the researcher monitoring participants’ progress via the app backend. The ES and HIIT groups’ intervention involved simple bodyweight exercises that included activities such as jumping jacks, lateral jumps, high-knee taps, high-knee with claps, and bodyweight squats, with each exercise lasting 1 min.

The ES group has a total weekly exercise duration of 75 min. Participants completed 11 min of exercise per day, Monday through Friday, and 10 min per day on weekends. Exercise bouts were distributed intermittently between 07:00 and 22:00, each lasting ≤ 3 min and separated by at least 60 min. This protocol aligns with the current operational definition of “exercise snacks,” which are described as isolated bouts of vigorous exercise lasting ≤5 min and performed periodically throughout the day (27).

The HIIT group has a total weekly training duration of 75 min, matching that of the ES group. Participants trained three times per week, with each session lasting 25 min and including five sets of each exercise (e.g., jumping jacks, lateral jumps, high-knee taps, high-knee with claps, and bodyweight squats), in a combination of repetitions. Each exercise set will last 5 min, interspersed with 3 min of rest.

**Figure 2:**
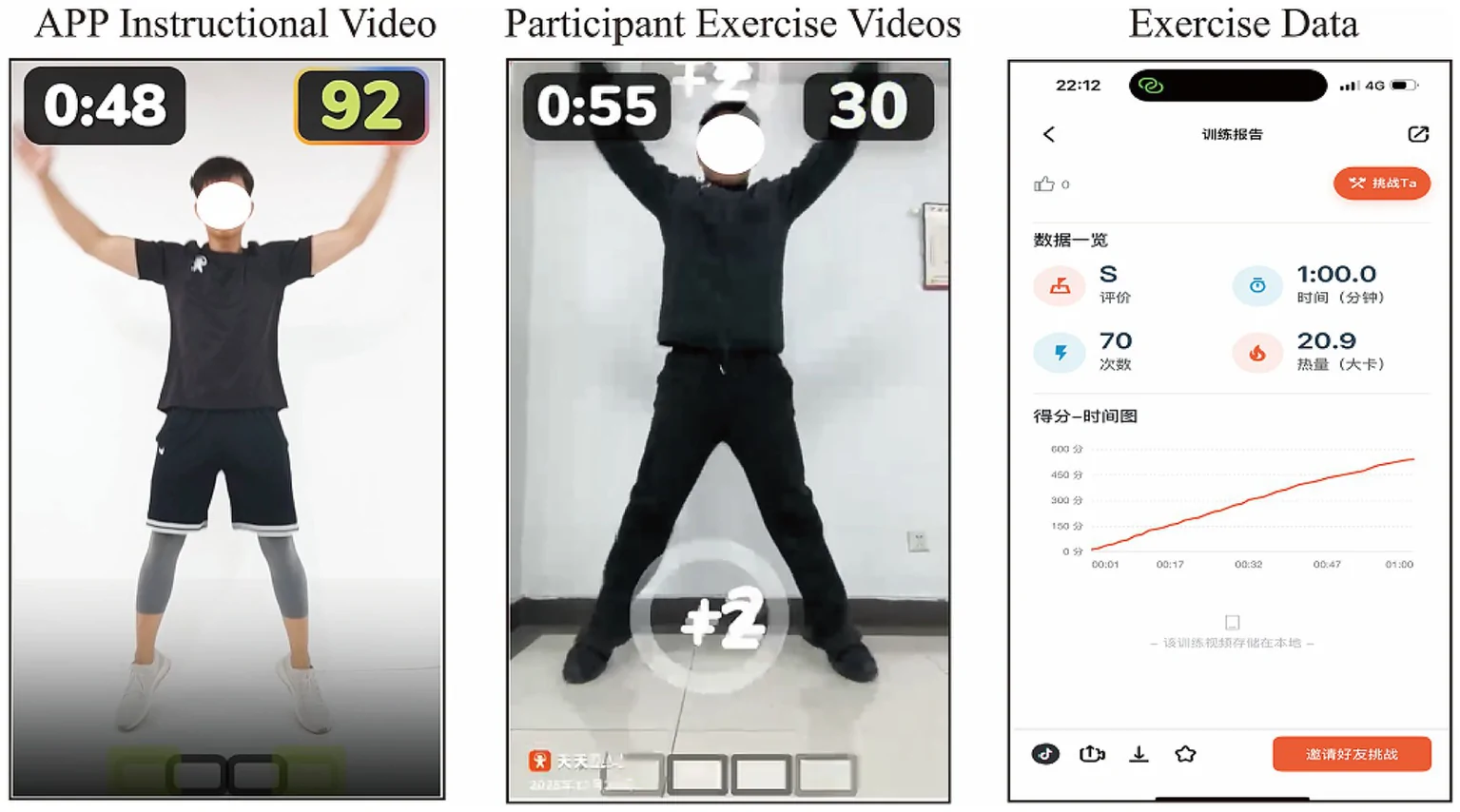
Exercise intervention process diagram.

During the intervention, training intensity was monitored using the Borg CR-10 rating of perceived exertion (RPE) scale. The target exercise intensity was operationally defined as hard or higher perceived exertion, corresponding to an RPE ≥ 6 on the Borg CR-10 scale (28). Participants reported their RPE immediately after each ES bout or HIIT session, and weekly mean RPE values were calculated for each participant as a process-related indicator to compare the perceived exertional demand between the ES and HIIT exercise formats throughout the 8-week intervention. Exercise quality was monitored using the smart exercise app. Participants were required to achieve an “S” rating (Figure 2), which was generated primarily based on the number of completed repetitions and movement quality relative to the standardized demonstration. Because this rating was not derived from heart-rate or wearable-device data, it was used as a practical indicator of movement quality and task completion.

Before the intervention, all participants attended a standardized educational session to familiarize them with the Borg CR-10 RPE scale and the talk test. The talk test was used as a complementary practical criterion for vigorous-intensity exercise, whereby participants were expected to be able to speak only short phrases of approximately two words while exercising. To standardize implementation, each participant completed two supervised familiarization exercise bouts under the guidance of a sports medicine specialist before beginning the prescribed intervention.

### 2.4 Outcomes measurements

#### 2.4.1 Body composition

We utilized the InBody 270 (InBody Co., Ltd., Seoul, Korea) to evaluate participants’ body weight, body mass index (BMI), and body fat percentage (PBF). This device employs bioelectrical impedance analysis (BIA) technology, measuring impedance at 20 kHz and 100 kHz frequencies via four-pole, eight-point tactile electrodes. Participants fasted for at least 2 h before testing, avoided strenuous exercise, emptied their bladders, and removed all metal jewelry. During testing, participants stood barefoot on the platform, held the handle electrodes with both hands, and maintained the standard posture for approximately 15 s to complete the measurement.

#### 2.4.2 Cardiometabolic health

To assess participants’ cardiometabolic health, we measured blood pressure (BP), serum high-density lipoprotein cholesterol (HDL-C), low-density lipoprotein cholesterol (LDL-C), total cholesterol (TC), and triglycerides (TG). Systolic and diastolic BP (SBP and DBP) were recorded with an automated BP monitor (Easy X 800, Jawon Medical Co., Kyungsan, Korea) after at least 5 min of seated rest. For biochemical assays (HDL-C, LDL-C, TC, TG), ≥5 mL of venous blood was drawn from the brachial vein using a disposable syringe after an overnight fast of at least 9 h; samples were centrifuged for ~10 min at 3000 rpm to separate serum, stored at low temperature, and sent to a certified laboratory for analysis. Pre-test (the day before training) and post-test (the day after training) samples were collected in the morning (29).

#### 2.4.3 Lower-body explosive power

##### 2.4.3.1 Standing long jumps (cm)

The standing long jumps (SLJ) test required participants to stand with their feet shoulder-width apart behind a starting line and their arms loosely hanging at their sides. Participants executed a countermovement with their legs and arms and jumped with maximal effort in the horizontal direction. Participants had to land with both feet simultaneously and could not fall forward or backward. The horizontal distance between the starting line and the heel of the rear foot was measured using a tape measure to the nearest 1 cm (30, 31). Each participant performed three trials, and the best (maximum) distance was recorded for analysis (31). SLJ distance reliability across three measurements: ICC = 0.993.

#### 2.4.4 Cardiorespiratory fitness

Participants ran on a 400-metre round track for a total duration of 12 min. They were highly motivated to run as many laps as possible. The total number of laps was counted, and the finishing point was marked. Total distance (in metres) covered in 12 min was calculated by multiplying the number of complete laps by 400 plus the distance covered (in metres) in the final incomplete lap. The distance in metres was converted into km, and the following equation was used to predict the V̇O_2max_ (32).

$$\;\dot{V}O_{2\max}\;(\mathrm{ml}\cdot kg^{-1}\cdot \mathrm{mi}n^{-1})=(\begin{array}{l} 22.351\times \text{distance}\;\\ \text{covered in kilometres} \end{array})-11.288$$

The validity of the Cooper 12-min run test has been confirmed, with a correlation coefficient of 0.897 between field-test distance and laboratory-determined V̇O_2max_ (32).

#### 2.4.5 Diet record

A diet diary (dietary record) was provided during the first and last week of the study to perform a quantitative analysis of the diets. This method consisted of asking the participants to write down, daily, the food they ingested during those days. The intake of each participant was calculated by averaging the 7 days, using the Bohe Health App.

### 2.5 Statistical analysis

No missing data were observed for the analyzed outcomes. Outliers were screened before analysis using boxplots and standardized residuals, and no data points were excluded. Data were presented as mean ± standard deviation (SD). Data normality was assessed using the Shapiro–Wilk test. The effects of time, group, and their interaction on each outcome were examined using a two-way repeated-measures analysis of variance (ANOVA), with time as the within-subject factor and group as the between-subject factor. Main effects of time and group, as well as the time × group interaction, were reported for each outcome. When a significant time × group interaction was observed, Bonferroni-adjusted simple effects analyses were performed to identify within-group changes over time and between-group differences at each time point. When the interaction was not significant, the main effects of time and group were interpreted to describe the overall effects.

Repeated-measures data were assessed using Mauchly’s test of sphericity; when the assumption of sphericity was violated, the Greenhouse–Geisser correction was applied. Partial eta-squared ( $\eta_{p}^{2}$ ) was used as the effect size for ANOVA main effects and interaction effects, with effect sizes categorized as trivial ( $\eta_{p}^{2}$ < 0.01), small (0.01 ≤ $\eta_{p}^{2}$ < 0.06), moderate (0.06 ≤ $\eta_{p}^{2}$ < 0.14), and large ( $\eta_{p}^{2}$ ≥ 0.14). Cohen’s d was used to quantify the magnitude of pairwise comparisons in *post hoc* or simple effects analyses, with d < 0.5, 0.5 ≤ d < 0.8, and d ≥ 0.8 considered small, moderate, and large effects, respectively (32). Statistical significance was set at *p* < 0.05. All analyses were performed using SPSS statistical software, version 25.0 (IBM Corp., Armonk, NY, United States).

## 3 Results

**Figure 3:**
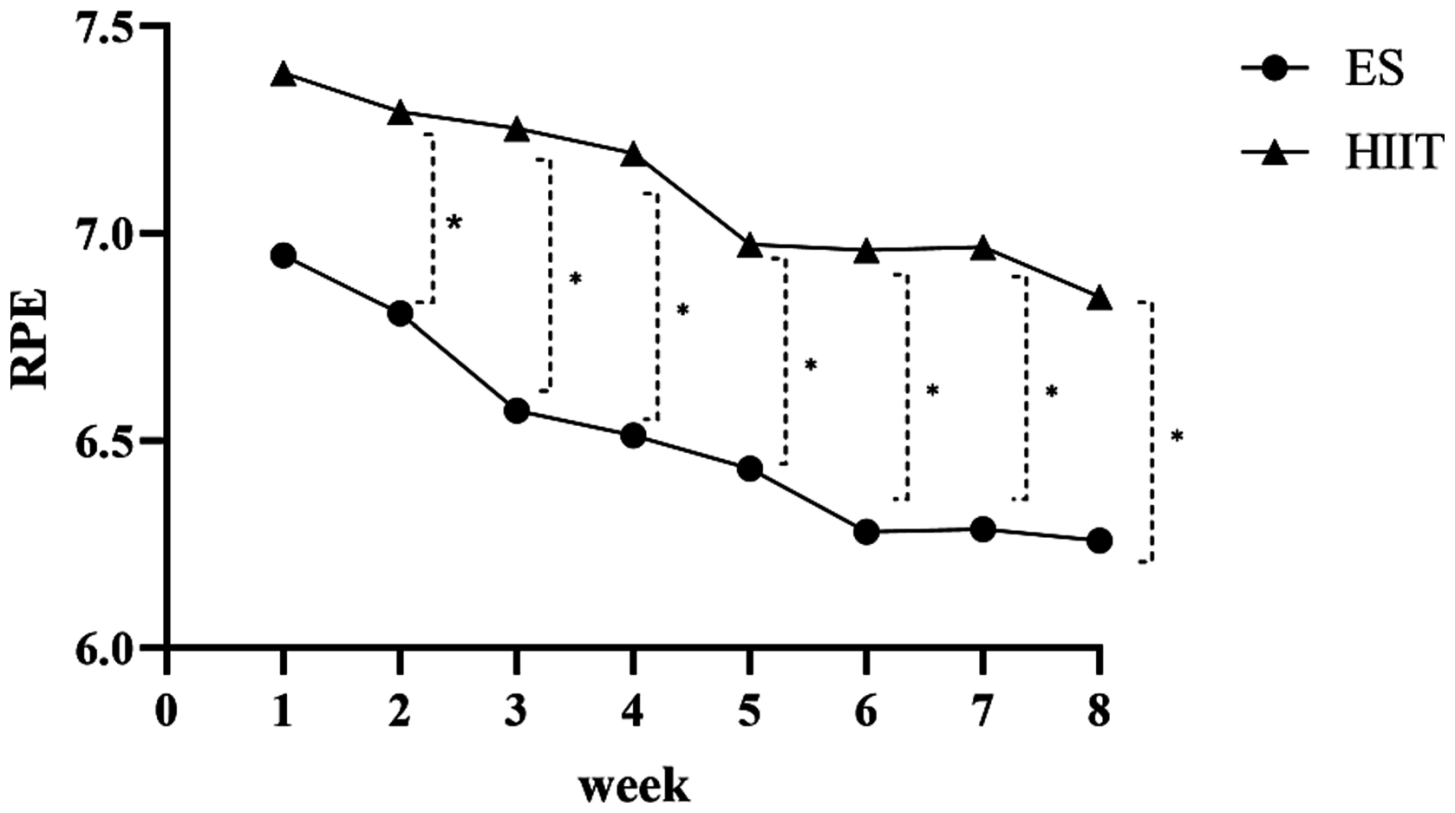
RPE data remotely collected through the online platform throughout the intervention. ES, exercise snacks group; HIIT, high-intensity interval training; *, Significant differences were found between the ES and HIIT groups (*p* < 0.05).

No participants withdrew from the ES and HIIT groups. The ES and HIIT groups had 100% compliance. Baseline anthropometric variables did not differ between groups (*p* > 0.05). The participants’ calorie intake during the first and eighth weeks is shown in Table 1. The findings highlighted a statistically insignificant difference between the groups before and after the intervention (*p* > 0.05) (Table 1). As a process-related indicator of perceived exertional demand, weekly mean RPE was significantly lower in the ES group than in the HIIT group throughout the intervention period (Figure 3).

**Table 1:** Baseline characteristics and estimated daily energy intake.

Variable	ES	HIIT	*p*
Age	20.98 ± 0.43	21.03 ± 0.44	0.74
Height (cm)	161.06 ± 6.64	160.32 ± 6.25	0.76
Weight (kg)	73.55 ± 12.84	69.15 ± 7.90	0.27
BMI (kg/m^2^)	28.21 ± 3.43	26.87 ± 2.35	0.22
Body fat percentage (%)	39.35 ± 5.30	36.71 ± 4.28	0.15
Cooper 12-min run test (V̇O_2max_)	29.85 ± 6.26	34.08 ± 7.82	0.25
Pre estimated daily energy intake (kcal/d)	1227.68 ± 153.23	1286.00 ± 177.36	0.18
Post estimated daily energy intake (kcal/d)	1220.99 ± 191.87	1298.61 ± 198.56	0.13

ES: Exercise Snacks; HIIT: High-Intensity Interval Training; BMI: Body Mass Index.

### 3.1 Body composition

#### 3.1.1 Body weight (kg)

**Figure 4:**
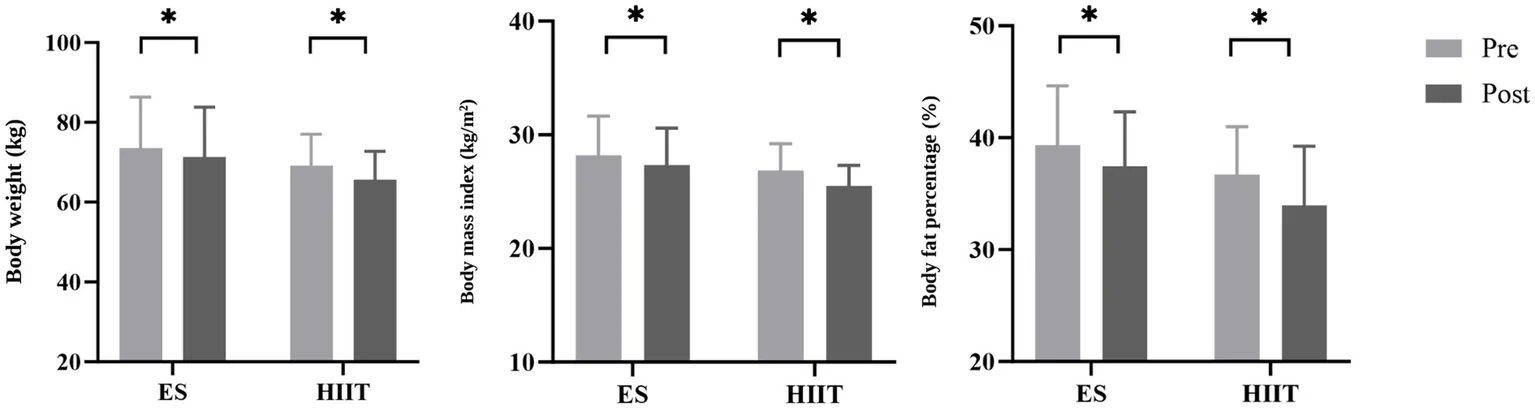
Effects of exercise snacks on body composition. ES, exercise snacks group; HIIT, high-intensity interval training; *, indicates a significant difference from baseline (*p* < 0.05). BF%, body fat percentage; BMI, body mass index.

A non-significant interaction effect between time and group was observed (*F* = 1.86, *p* = 0.18, $\eta_{p}^{2}$ =0.06). The time main effects analysis showed a significant decrease (*F* = 41.56, *p* < 0.001, $\eta_{p}^{2}$ =0.59). The group main effects analysis showed a non-significant difference (*F* = 1.76, *p* = 0.18, $\eta_{p}^{2}$ =0.06) (Figure 4; Table 1).

#### 3.1.2 Body mass index (BMI, kg/m^2^)

A non-significant interaction effect between time and group was observed (*F* = 1.99, *p* = 0.17, $\eta_{p}^{2}$ =0.07). The time main effects analysis showed a significant decrease (*F* = 41.41, *p* < 0.001, $\eta_{p}^{2}$ =0.60). The group main effects analysis showed a non-significant difference (*F* = 2.50, *p* = 0.12, $\eta_{p}^{2}$ =0.08) (see Table 2).

**Table 2:** Effects of exercise snacks on body composition, cardiometabolic health, cardiorespiratory fitness, and lower-body explosive power.

Variable	Group	PRE	POST	Cohen’s d (95% CIs)		RM ANOVA			
				Within-group	Between-group	Source	*F*	*p*	*η2*
Body composition	Body weight (kg)					Time × group	1.86	0.184	0.06
	ES	73.55 ± 12.84	71.33 ± 12.49	1.02 (0.38 to 1.64)	0.52 (−0.18 to 1.28)	Time	41.56	<0.001	0.6
	HIIT	69.15 ± 7.9	65.73 ± 7.06	1.31 (0.60 to 2.00)		group	1.76	0.196	0.06
	Body mass index (BMI, kg/m^2^)					Time × group	1.99	0.169	0.07
	ES	28.21 ± 3.43	27.34 ± 3.24	0.99 (0.36 to 1.61)	0.70 (−0.05 to 1.43)	Time	41.42	<0.001	0.6
	HIIT	26.87 ± 2.35	25.51 ± 1.80	1.34 (0.62 to2 0.03)		group	2.5	0.125	0.08
	Body fat percentage (%)					Time × group	0.8	0.38	0.03
	ES	39.35 ± 5.30	37.46 ± 4.85	1.12 (0.46 to 1.76)	0.69 (−0.06 to 1.42)	Time	23.71	<0.001	0.46
	HIIT	36.71 ± 4.28	33.97 ± 5.27	0.84 (0.24 to 1.42)		group	3.1	0.09	0.1
Lower-body explosive power	Standing long jumps					Time × group	0.19	0.669	0.007
	ES	1.49 ± 0.19	1.61 ± 0.11	0.79 (0.20 to 1.36)	0.36 (−0.36 to 1.08)	Time	21.26	<0.001	0.43
	HIIT	1.56 ± 0.14	1.66 ± 0.15	0.95 (0.32 to 1.56)		group	1.44	0.24	0.05
Cardiorespiratory fitness	Cooper 12-min run test (V̇O_2max_)					Time × group	0.17	0.684	0.006
	ES	29.85 ± 6.26	35.85 ± 7.28	1.17 (0.49 to 1.82)	0.43 (−0.30 to 1.15)	Time	24.29	<0.001	0.47
	HIIT	34.08 ± 7.82	39.16 ± 8.08	0.72 (0.14 to 1.28)		group	2.36	0.136	0.08
Cardiometabolic health	TG (mmol/L)					Time × group	0.01	0.938	0
	ES	1.26 ± 0.76	1.25 ± 0.82	0.02 (−0.48 to 0.53)	0.47 (−0.26 to 1.19)	Time	0.003	0.959	0
	HIIT	0.95 ± 0.52	0.95 ± 0.45	0.01 (−0.50 to 0.51)		group	1.77	0.194	0.06
	SBP (mmHg)					Time × group	0.55	0.467	0.02
	ES	115.13 ± 8.21	110.66 ± 6.99	0.87 (0.26 to 1.46)	0.09 (−0.63 to 0.80)	Time	32.26	<0.001	0.54
	HIIT	115.87 ± 5.36	110.07 ± 6.74	1.22 (0.53 to 1.88)		group	0.001	0.978	0
	DBP (mmHg)					Time × group	6.63	0.016	0.19
	ES	72.13 ± 7.25	72.40 ± 7.6	0.05 (−0.46 to 0.55)	0.35 (−0.35 to 1.06)	Time	5.4	0.03	0.16
	HIIT	75.33 ± 6.57	70.13 ± 5.32	0.92 (0.30 to 1.52)		group	0.05	0.835	0.002
	HDL (mmol/L)					Time × group	0.06	0.818	0.002
	ES	1.28 ± 0.27	1.22 ± 0.26	0.49 (−0.06 to 1.02)	0.29 (−0.43 to 1.01)	Time	2.96	0.1	0.09
	HIIT	1.34 ± 0.29	1.29 ± 0.22	0.22 (−0.29 to 0.73)		group	0.49	0.492	0.02
	TC (mmol/L)					Time × group	0.24	0.628	0.008
	ES	3.98 ± 0.59	3.72 ± 0.58	0.68 (0.10 to 1.23)	0.03 (−0.69 to 0.74)	Time	9.64	0.004	0.26
	HIIT	3.89 ± 0.75	3.7 ± 0.67	0.46 (−0.08 to 0.99)		group	0.06	0.819	0.002
	LDL (mmol/L)					Time × group	0.05	0.836	0.002
	ES	2.55 ± 0.52	2.32 ± 0.57	0.70 (0.12 to 1.25)	0.75 (−0.64 to 0.79)	Time	12.21	0.002	0.31
	HIIT	2.48 ± 0.75	2.28 ± 0.63	0.58 (0.03 to 1.13)		group	0.07	0.794	0.002

ES, exercise snacks; HIIT, high-intensity interval training; BMI, body mass index; V̇O_2max_, maximal oxygen uptake; SLJ, standing long jump; TG, triglycerides; SBP, systolic blood pressure; DBP, diastolic blood pressure; HDL-C, high-density lipoprotein cholesterol; TC, total cholesterol; LDL-C, low-density lipoprotein cholesterol.

#### 3.1.3 Body fat percentage (%)

A non-significant interaction effect between time and group was observed (*F* = 0.80, *p* = 0.38, $\eta_{p}^{2}$ =0.03). The time main effects analysis showed a significant decrease (*F* = 23.71, *p* < 0.001, $\eta_{p}^{2}$ =0.46). The group main effects analysis showed a non-significant difference (*F* = 3.10, *p* = 0.09, $\eta_{p}^{2}$ =0.10).

### 3.2 Lower-body explosive power

#### 3.2.1 Standing long jumps

**Figure 5:**
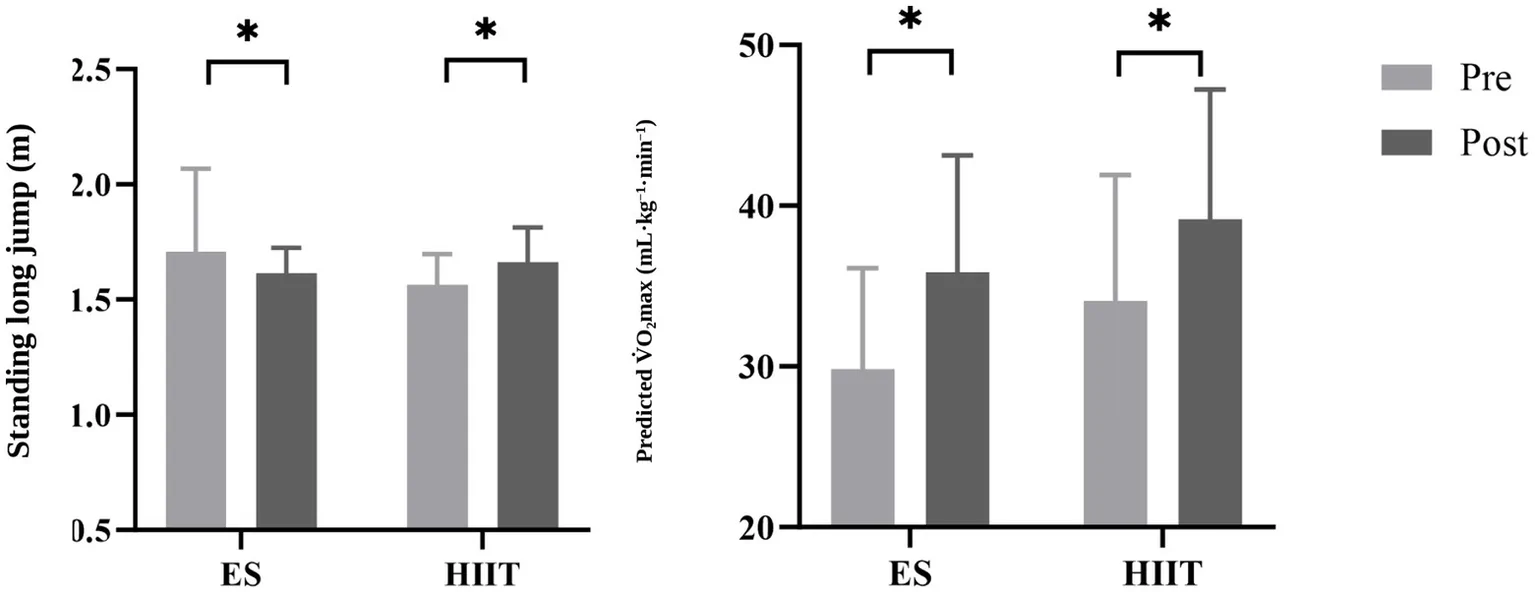
Effects of exercise snacks on lower-body explosive power and V̇O_2max_. ES, exercise snacks group; HIIT, high-intensity interval training; *, Indicates a significant difference from baseline (*p* < 0.05). V̇O_2max_, maximal oxygen uptake.

A non-significant interaction effect between time and group was observed (*F* = 0.19, *p* = 0.67, $\eta_{p}^{2}$ =0.01). The time main effects analysis showed a significant increase (*F* = 21.56, *p* < 0.001, $\eta_{p}^{2}$ =0.43). The group main effects analysis showed a non-significant difference (*F* = 1.44, *p* = 0.24, $\eta_{p}^{2}$ =0.05) (Figure 5).

### 3.3 V̇O₂_max_

A non-significant interaction effect between time and group was observed (*F* = 0.17, *p* = 0.68, $\eta_{p}^{2}$ =0.01). The time main effects analysis showed a significant increase (*F* = 24.29, *p* < 0.001, $\eta_{p}^{2}$ =0.47). The group main effects analysis showed a non-significant difference (*F* = 2.36, *p* = 0.14, $\eta_{p}^{2}$ =0.08) (Figure 5).

### 3.4 Cardiometabolic health

#### 3.4.1 TG (mmol/L)

**Figure 6:**
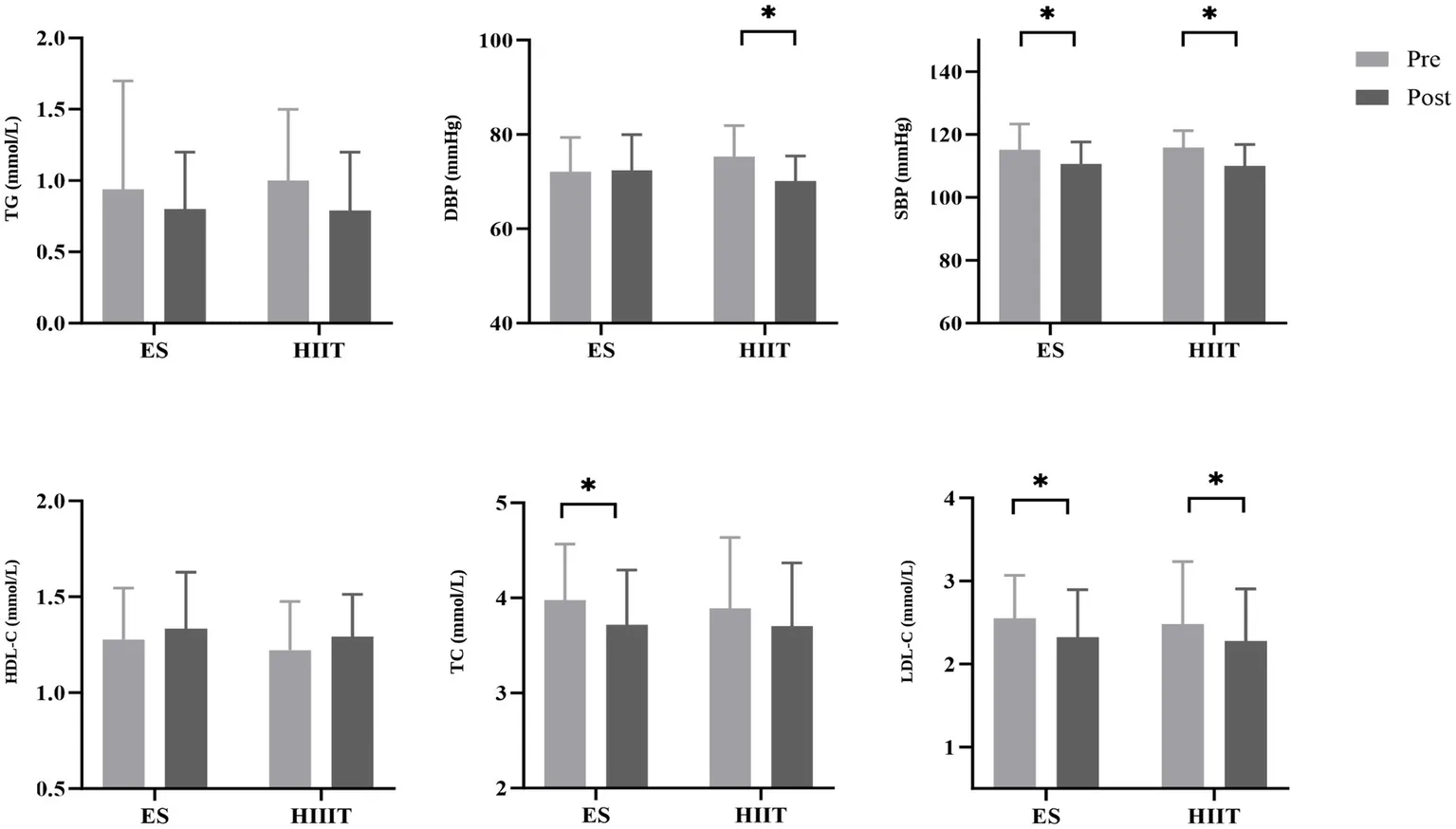
Effects of exercise snacks on cardiometabolic health. ES, exercise snacks group; HIIT, high-intensity interval training; *, indicates a significant difference from baseline (*p* < 0.05). TG, triglycerides; SBP, systolic blood pressure; DBP, diastolic blood pressure; HDL-C, high-density lipoprotein cholesterol; TC, total cholesterol; LDL-C, low-density lipoprotein cholesterol.

A non-significant interaction effect between time and group was observed (*F* = 0.01, *p* = 0.94, $\eta_{p}^{2}$ =0.001). The time main effects analysis showed no significant change (*F* = 0.003, *p* = 0.96, $\eta_{p}^{2}$ =0.001). The group main effects analysis showed no significant difference (*F* = 1.78, *p* = 0.19, $\eta_{p}^{2}$ =0.06) (Figure 6).

#### 3.4.2 SBP (mmHg)

A non-significant interaction effect between time and group was observed (*F* = 0.54, *p* = 0.48, $\eta_{p}^{2}$ =0.02). The time main effects analysis showed a significant decrease (*F* = 32.26, *p* < 0.001, $\eta_{p}^{2}$ =0.54). The group main effects analysis showed a non-significant difference (F = 0.54, *p* = 0.47, $\eta_{p}^{2}$ =0.02).

#### 3.4.3 DBP (mmHg)

A significant interaction effect between time and group was observed (*F* = 6.63, *p* = 0.02, $\eta_{p}^{2}$ =0.19). After the training period, significantly decrease in the HIIT group (*p* = 0.002, d = 0.86), and non-significant decrease were noted in the ES groups (*p* = 0.86, d = −0.04). Non-significant between-group difference was observed (*p* = 0.35, d = 0.35).

#### 3.4.4 HDL-C (mmol/L)

A non-significant interaction effect between time and group was observed (*F* = 0.05, *p* = 0.82, $\eta_{p}^{2}$ =0.002). The time main effects analysis showed a non-significant increase (*F* = 2.97, *p* = 0.10, $\eta_{p}^{2}$ =0.10). The group main effects analysis showed no significant difference (*F* = 0.49, *p* = 0.49, $\eta_{p}^{2}$ =0.12).

#### 3.4.5 TC (mmol/L)

A non-significant interaction effect between time and group was observed (*F* = 0.24, *p* = 0.63, $\eta_{p}^{2}$ =0.01). The time main effects analysis showed a significant decrease (*F* = 9.64, *p* = 0.004, $\eta_{p}^{2}$ =0.26). The group main effects analysis showed no significant difference (F = 0.05, *p* = 0.82, $\eta_{p}^{2}$ =0.002).

#### 3.4.6 LDL-C (mmol/L)

A non-significant interaction effect between time and group was observed (*F* = 0.04, *p* = 0.84, $\eta_{p}^{2}$ =0.002). The time main effects analysis showed a significant decrease (*F* = 12.01, *p* = 0.002, $\eta_{p}^{2}$ =0.30). The group main effects analysis showed no significant difference (*F* = 0.07, *p* = 0.79, $\eta_{p}^{2}$ =0.002).

## 4 Discussion

To our knowledge, this is the first study to examine, in a real-world setting, the effects of ES versus HIIT on body composition, cardiometabolic health, V̇O_2max_, and lower-body explosive power in overweight and obese female college students. Preliminary findings from this exploratory trial suggest that the weekly mean RPE was significantly lower in the ES group than in the HIIT group throughout the intervention period. Both the ES and HIIT groups showed significant pre-post improvements in body composition, V̇O₂_max_, lower-body explosive power, and selected cardiometabolic markers (SBP, TC, LDL-C). No significant changes were observed in TG, HDL-C, and DBP.

This study showed that after 8 weeks, both the ES and HIIT groups achieved significant pre–post reductions in weight, BMI, and body fat percentage. These findings are consistent with those of Zhou et al. (11), who found significant reductions in weight and body fat percentage in obese females following 8 weeks of multiple short-duration exercise sessions in a real-world setting. However, the findings of the current study do not support the previous research. Wan’s et al. (33) meta-analysis found that, compared to the control group, the implementation of ES did not result in significant changes in body fat percentage or body weight. Similarly, Sultana’s et al. (34) systematic review highlighted the limited effectiveness of short-duration, high-intensity exercise interventions in optimizing body composition. This inconsistency may be attributed to insufficient exercise dosage in the included studies (e.g., three daily 60-s stair sprints) and to variability in intervention duration (ranging from 4 to 8 weeks) (33, 34). This study met the WHO guideline of 75 min of high-intensity activity per week (4), roughly 10–11 min daily, which is likely adequate to achieve significant improvements in body composition. Another possible explanation is that individuals with overweight or obesity generally have higher baseline body weight and adiposity, leaving greater potential for measurable reductions after exercise training. Westerterp (35) reported that exercise training may produce more pronounced improvements in body composition in this population. Therefore, the significant reductions in body weight and body fat percentage observed in the present study and in Zhou et al. (11) may be partly attributable to the greater adaptive potential of overweight or obese participants.

This study showed that after 8 weeks, both the ES and HIIT groups achieved significant pre–post improvements in lower-body explosive power and CRF. This is consistent with previous laboratory studies (13, 14), in which inactive or sedentary adults performed three daily exercise snacks, 1-4 h apart, using stair climbing or cycling sprints, thrice weekly for six weeks. These supervised exercise snack interventions led to modest (~4–5%) improvements in CRF compared with baseline or a non-exercising control group (13, 14). Zhu’s et al. (36) study also reported that after 6 weeks of performing ES three times weekly for 30–60 s “as fast as possible,” significant improvements in peak torque were observed during both 30°/s isometric and 300°/s isokinetic strength tests. In contrast to Babir et al. (18), who reported no improvements in CRF (V̇O₂_peak_ and peak power) following remotely supervised bodyweight ES in real-world settings. Similarly, Sperlich et al. (37) observed no increase in V̇O₂_peak_ after a 4-week home-based ES intervention. The discrepancy may be attributable to the difficulty of achieving sufficient intensity with bodyweight ES in real-world settings. For instance, Sperlich et al. (37) reported that during a 6-min video-guided bodyweight training session, participants’ average heart rate remained below 75% of their maximal heart rate despite being instructed to exercise at maximal effort. In this study, each session required participants to reach an S-level rating, and overall RPE scores remained between 6 and 7. Another possible explanation is that the low exercise dose in the Fiona et al. (18), protocol (three sessions per week, three bouts ≤1 min each) may have been insufficient to elicit statistically significant adaptations.

This study found that both the ES and HIIT groups showed no significant improvements in TG and HDL-C levels. This aligns with Ke-wen’s meta-analysis, which also reported no significant effects of ES on these measures compared to control conditions (33). Although ES may exert a limited influence on TG and HDL-C, positive effects have been observed on other lipid profile parameters (TC, LDL-C). Brief high-intensity exercise may enhance lipoprotein lipase (LPL) activity, facilitating TG hydrolysis and LDL-C clearance (38–40). Previous studies indicate that ES can improve insulin sensitivity promotes skeletal muscle glucose uptake, suppresses hepatic gluconeogenesis and glycogenolysis, and reduces cholesterol synthesis (41–44). Increased insulin responsiveness augments the utilization of fatty acids as an energy substrate, lowering circulating free fatty acid concentrations (38, 45). Due to the lack of direct measurements of these mechanistic biomarkers in this study, these explanations should be considered speculative. Future studies should include relevant physiological and biochemical markers to clarify the mechanisms underlying changes in lipid profiles following ES and HIIT.

This study found that both the ES and HIIT groups experienced significant pre–post improvements in SBP, consistent with Chen et al.’s meta-analysis (46), which reported beneficial effects of ES on SBP. Similarly, Reljic et al. (47) observed that, following a 12-week biweekly cycling intervention, 65 inactive obese participants in the intervention group demonstrated modest improvements in mean arterial blood pressure. DBP appeared to respond differently to the two exercise formats. The lack of a significant DBP reduction following ES may be partly explained by participants’ baseline blood pressure being within the normotensive range, which limited the potential for further reductions. In addition, compared with HIIT, the ES protocol consisted of very brief bouts distributed across the day, which may have provided a less sustained cardiovascular stimulus for modifying diastolic pressure. Although a significant time × group interaction was observed for DBP, the post-intervention between-group difference was not significant. Therefore, the DBP result should be interpreted cautiously as a within-group differential rather than a between-group significant difference. Similar studies in normotensive populations have also reported limited or non-significant changes in DBP following exercise interventions (48, 49).

This study found that, under real-world conditions, no significant between-group differences in body composition, V̇O₂_max_, lower-body explosive power, SBP, TC, or LDL-C were observed between the ES and HIIT groups. This finding suggests that, when the weekly exercise dose was matched, both ES and HIIT were associated with significant improvements in these outcomes. In addition, weekly mean RPE was significantly lower in the ES group than in the HIIT group throughout the intervention period. This may be partly explained by the distributed structure of ES, in which brief bouts were spaced throughout the day, potentially reducing fatigue accumulation compared with more concentrated HIIT sessions. This finding may have important practical implications, as ES could provide a more flexible exercise option for individuals with limited time or difficulty completing longer sessions. However, given the small sample size, this finding should be interpreted cautiously.

## 5 Limitations

This study has several limitations. First, the sample size was small, and all participants were female, which may have reduced statistical power and limited the generalizability of the findings. Second, exercise intensity was not objectively quantified using heart rate, oxygen uptake, or blood lactate. The intensity classified by RPE and the talk test may not necessarily reflect true physiological high intensity and should be interpreted with caution. Third, body composition and V̇O₂_max_ were assessed using BIA and the Cooper 12-min run test, respectively, which may be less accurate than gold-standard methods such as dual-energy X-ray absorptiometry and laboratory-based gas analysis. Fourth, although blood pressure and lipid profiles were assessed, glucose metabolism indicators, such as fasting glucose, insulin, and HOMA-IR, were not included, which may have limited the comprehensiveness of the cardiometabolic assessment. Fifth, because the intervention was conducted in a real-world remote setting, adherence and exercise execution may have varied across participants. In addition, physical activity and sedentary behavior during the intervention were not objectively monitored using accelerometers. Future studies using accelerometers may help better determine whether ES and HIIT promote a more active lifestyle outside the prescribed intervention.

## 6 Conclusion

In conclusion, this study suggests that 75 min/week of bodyweight-based ES may be a practical exercise strategy for improving body composition, V̇O₂_max_, lower-body explosive power, and selected cardiometabolic markers (SBP, TC, LDL-C) in overweight and obese female college students. However, no significant change was observed in TG, HDL-C, or DBP. Given the small sample size, absence of a non-exercise control group, and limited assessment of metabolic indicators in the present study, further randomized controlled trials with larger samples and more comprehensive outcome measures are needed before ES can be promoted as an alternative exercise strategy.

## Data Availability

The raw data supporting the conclusions of this article will be made available by the authors, without undue reservation.
