# Correction: Effects of 75 min of weekly exercise snacks on body composition, cardiometabolic health, cardiorespiratory fitness, and lower-body explosive power in overweight and obese female university students

**DOI:** 10.3389/fpubh.2026.1940473

**Published:** 2026-09-01

**Authors:** Naijing Jin, Xin Zheng, Ziren Zhao, Fangyuan Zhao, Zhangyuting Shang, Kaixiang Zhou

**Affiliations:** 1 Journal Editorial Department, China Institute of Sport Science, Beijing, China; 2 College of Physical Education and Health Science, Chongqing Normal University, Chongqing, China; 3 Sports Coaching College, Beijing Sport University, Beijing, China

**Keywords:** body composition, exercise snacks, female, high-intensity interval training, overweight and obese

Authors Naijing Jin and Xin Zheng were erroneously assigned as equal contributing authors sharing first authorship. The equal contribution phrase has been removed.

The **Ethics statement** erroneously mentioned the ethics review institution as “Ethics Committee of Sports Science Experiment of Beijing Sport University.” The correct **Ethics statement** is:

“The studies involving humans were approved by the Ethics Committee of China Institute of Sport Science. The studies were conducted in accordance with the local legislation and institutional requirements. The participants provided their written informed consent to participate in this study.”

The name of the ethics review institution was incorrectly stated as “Beijing Sport University.” The correct name of the institution is “China Institute of Sport Science.” The ethics approval number remains unchanged.

A correction has been made to “Section **2 Materials and Methods**, subsection *2.1 Participants*, paragraph 2:

“The study was approved by the Ethics Committee of China Institute of Sport Science (No. 20250408), and all procedures were conducted in accordance with the Declaration of Helsinki.”

The original version of this article has been updated.

